# Poor Glycemic Control in Type 2 Diabetes Mellitus Patients in Two Tertiary Care Centers during COVID-19 Lockdown: A Descriptive Cross-sectional Study

**DOI:** 10.31729/jnma.6998

**Published:** 2022-03-31

**Authors:** Jagatkiran Oli, Ved Prakash Pant, Apeksha Niraula, Madhab Lamsal

**Affiliations:** 1 B.P. Koirala Institute of Health Sciences, Dharan, Sunsari, Nepal; 2 Department of Clinical Biochemistry, Tribhuvan University Teaching Hospital, Institute of Medicine, Maharajgunj, Kathmandu, Nepal

**Keywords:** *COVID-19*, *glycemic control*, *healthy lifestyle*, *lockdown*, *type 2 diabetes*

## Abstract

**Introduction:**

Lockdown enforced to control the rapid transmission of novel coronavirus has resulted in the confinement of people in the home and restrictions of movement. This may have altered the lifestyle and glycemic control of ype 2 diabetes mellitus patients. This study aimed to find the prevalence of poor glycemic control in type 2 diabetes mellitus patients in two tertiary care centres during COVID-19 lockdown.

**Methods:**

A descriptive cross-sectional study was conducted among 259 type 2 diabetes mellitus patients in selected hospitals from 1^st^ September to 30^th^ September 2020 after receiving ethical approval from the Departmental Research Unit, Biochemistry under Institutional Review Committee (Reference number: DRU/01/2020). A convenience sampling method was used. Data analysis was done by using Statistical Package for the Social Sciences version 26.0. Point estimate at 95% Confidence Interval was calculated along with frequency and proportion for binary data.

**Results:**

Among 259 patients with type 2 Diabetes Mellitus, 183 (70.65%) (65.10-76.20 at 95% Confidence Interval) had poor glycemic control during the lockdown period. Mean fasting and post-prandial blood glucose among these patients were 164.16±49.30 mg/dl and 246.76±69.86 mg/ dl respectively.

**Conclusions:**

Our study depicts that the majority of the type 2 diabetes mellitus patients had poor glycemic control during the lockdown period which was similar when compared to other studies.

## INTRODUCTION

The Corona Virus disease (COVID-19) was first noticed in Wuhan, China, on 31^st^ December 2019 was declared pandemic on 11^th^ March 2020 by World Health Organization (WHO).^1^ With the confirmation of the first case in Nepal on 23^rd^ January 2020,^2^ and the alarming rate of increasing COVID-19 patients in neighbouring countries China and India, the Government of Nepal imposed a nationwide lockdown on 24^th^ March 2020.^3^

Although lockdown effectively reduces the incidence and mortality of COVID-19,^4^ it has brought in the confinement of people in the home, restrictions of movement, shortage of food and medication supply, an economic burden which may have altered the patients' dietary habits, physical activity, compliance with drugs, a regular visit to doctor and blood glucose monitoring.

This study aimed to find out the prevalence of poor glycemic control in Type 2 Diabetes Mellitus (T2DM) patients from two tertiary care centres of Nepal during the COVID-19 lockdown period.

## METHODS

A descriptive cross-sectional study was conducted on Type 2 DM patients from the out-patient department of Rapti Provincial Hospital, Dang and Mahakali Hospital, Kanchanpur, Nepal from 1^st^ September to 30^th^ September 2020 after obtaining the ethical approval from Departmental Research Unit (DRU), Biochemistry, under Institutional Review Committee (IRC), B.P. Koirala Institute of Health Sciences (Reference number: DRU/01/2020). The convenience sampling method was used to recruit the patients. The sampling frame was prepared by the names and contact numbers of diagnosed patients of T2DM who visited the hospital for follow-up during the study period.

The sample size was calculated by using the formula,

n = (Z^2^ × p × q) / e^2^

  = (1.96^2^ × 0.5 × 0.5) / (0.065)^2^

  = 228

Where,

n = minimum required sample size
Z = 1.96 at 95% Confidence Interval (CI)
p = prevalence taken as 50% for maximum sample size
q = 1-p
e = margin of error, 6.5%

Adding 10% for non-response rate, the sample size of 253 was calculated. However, we collected data from 259 patients.

Adult patients diagnosed with Type 2 Diabetes Mellitus for at least ten months, under medication, patients with blood glucose reports tested during the study period and who gave consent were included. Suspected or diagnosed COVID-19 cases and patients with mental retardation, patients older than 80 years of age were excluded from the study. The total patients fulfilling the inclusion criteria were interviewed over the telephone after getting informed consent from the study participants.

A well-structured questionnaire was used that contained four sections. Section A contained the Sociodemographic profile of study participants; section B contained the Clinical and nutritional status of study participants; section C contained Global Physical Activity Questionnaire (GPAQ), and section D contained Perceived Dietary Adherence Questionnaire (PDAQ). Dietary adherence was assessed using the validated Perceived Dietary Adherence Questionnaire (PDAQ), a seven-point Likert scale-based tool to measure dietary compliance.^5^ It had nine questions with scores ranging from zero to seven. The total score was 63. A score greater than or equal to 75% of the total score was considered as high adherence, (50-75%) of the total as medium adherence and less than 50% of the total as low-adherence. For physical activity, the data was cleaned and analyzed as per GPAQ analysis guidelines.^7^ Total Metabolic Equivalents (METs) values of individuals were calculated in weeks and were converted into MET-minutes per week (MET-min/week). A MET score greater than or equal to 1500 MET min/week was considered as a high level of Physical Activity, (6001500) MET min/week was considered as moderate and less than 600 MET min/week as low level of Physical Activity.

Patients having fasting plasma glucose less than 130 mg/dl and postprandial plasma glucose less than 180 mg/dl were considered to have controlled blood glucose; else, they were said to have uncontrolled blood glucose.^8^

Data was entered in Microsoft Excel 2016 and analyzed in Statistical Package for the Social Sciences version 26.0. Point estimate at 95% Confidence Interval was calculated along with frequency and percentage for binary data.

## RESULTS

Among 259 patients with Type 2 Diabetes Mellitus, 183 (70.65%) (65.10-76.20 at 95% Confidence Interval) had poor glycemic control during the lockdown period. Mean fasting and post-prandial blood glucose among these patients were 164.16±49.30 mg/dl and 246.76±69.86 mg/dl respectively. Both mean fasting and postprandial blood glucose levels were higher in patients aged >55 years and urban population in comparison to patients aged ≤55 years and rural population respectively. Patients diagnosed with T2DM for <5 years had higher mean blood glucose levels than those for >5 years (Table 1).

**Table 1:** Glycemic status and associated variables of patients with poor glycemic control (n = 183).

Variables	Categories	Blood glucose level (Mean±SD)	
		Fasting	Post-prandial
Age	≤55 years	163.95±47.72	243.28±65.11
	>55 years	164.38±51.26	252.91 ±72.51
Sex	Male	164.10±46.85	248.20±69.90
	Female	164.29±54.64	247.11±66.67
Duration of T2DM	≤5 years	165.36±50.85	254.51 ±73.26
	>5 years	162.46±47.31	238.49±60.97
Residence	Rural	156.08±44.35	237.79±63.96
	Urban	169.40±51.78	254.39±71.14
BMI	Normal	162.92±50.65	246.15±74.71
	Overweight	167.16±48.39	254.83±69.75
	Obese	157.82±50.65	231.76±56.10

Among 183 T2DM patients with poor glycemic control, 125 (68.31%) were male, and 58 (31.69%) were female. The mean age of the patients was 55.56±10.82 years. The majority of them were married 174 (95.08%) and self-employed 72 (39.34%). Patients residing in the urban area were 111 (60.66%). The majority of the study participants 107 (58.47%) had a history of T2DM for ≤5 years and 99 (54.09%) were overweight. The most common comorbidity associated with T2DM was hypertension 50 (27.32%). Most patients took oral hypoglycemic agents 172 (93.99%), followed by oral hypoglycemic agents with insulin 7 (3.82%) (Table 2).

**Table 2:** Socio-demographic profile, clinical and nutritional status of patients with poor glycemic control (n= 183).

Characteristics	Category	n (%)
Age (in years)	≤55	96 (52.46)
	>55	87 (47.54)
Gender	Male	125 (68.31)
	Female	58 (31.69)
Marital status	Married	174 (95.08)
	Widow/Divorced	9 (4.92)
Occupation	Government Employed	24 (13.11)
	Self employed	72 (39.34)
	Retired	17 (9.29)
	Homemaker	46 (25.14)
	Unable to work	11 (6.01)
	Others	13 (7.10)
Educational	Informal education	51 (27.87)
status	Primary	40 (21.86)
	Secondary	43 (23.49)
	Higher secondary	21 (11.48)
	Graduate	18 (9.84)
	Post-graduate and above	10 (5.46)
Residence	Rural	72 (39.34)
	Urban	111 (60.66)
Years of T2DM	≤5 years	107 (58.47)
	>5 years	76 (41.53)
BMI	Normal	46 (25.14)
	Overweight	99 (54.09)
	Obese	38 (20.77)
Smoking habit	Always	10 (5.46)
	Frequently	5 (2.73)
	Occasional	23 (12.57)
	Used to smoke, later stopped	27 (14.75)
	Never	118 (64.48)
Drinking habit	Always	3 (1.64)
	Frequently	5 (2.73)
	Occasional	50 (27.32)
	Used to drink, later stopped	26 (14.21)
	Never	99 (54.09)
Comorbidities	Hypertension (HTN)	50 (27.32)
	Dyslipidemia	12 (6.56)
	HTN + Dyslipidemia	15 (8.19)
	HTN + Others	1 (0.55)
	Others	11 (6.01)
	None	94 (51.37)
Medications Taken	Oral hypoglycemic agents (OHA)	172 (93.99)
	Insulin	4 (2.19)
	OHA + Insulin	7 (3.82)

After considering only high adherence as adherence and medium and low adherence as non-adherence, we found 14 (7.7%) of patients were adherent to diet while most of them, 169 (92.3%) were non-adherent. Out of 183 patients with poor glycemic control, 79 (43.2%) had below the recommended physical activity level (Table 3).

**Table 3:** Distribution of patients with poor glycemic control according to adherence to diet and physical activity (n= 183).

Description	Categories	n (%)
	High	14 (7.65)
Dietary adherence	Medium	118 (64.48)
	Low	51 (27.87)
	High	44 (24.04)
Physical activity level	Moderate	60 (32.79)
	Low	79 (43.17)

Positive responses to Perceived Dietary Adherence Questionnaire (PDAQ) were that, in most of the days in a week, patients did not eat foods high in sugar (6.19±1.27), did not eat foods high in fat (5.37±1.95). However, patients rarely ate fish or foods rich in Omega-3 fatty acids (0.76±1.25) (Table 4).

**Table 4:** Response of patients with poor glycemic control to Perceived Dietary Adherence Questionnaire (PDAQ) (n= 183).

Questions: In last 7 days	Mean±SD (days in last one week)
How many days have you followed a healthful eating plan with appropriate serving sizes?	4.32±1.47
How many days did you eat an adequate number of fruit and vegetable servings?	4.42±2.08
How many days did you eat carbohydrate-containing foods with a low Glycemic Index? (Example: dried beans, lentils, barley, pasta, low-fat dairy products	3.38±2.22
How many days did you not eat foods high in sugar, such as cakes, cookies, desserts, candies?	6.19±1.27
How many days did you eat foods high in fibre, such as oatmeal, high fibre cereals, whole-grain bread?	5.36 ± 2.07
How many days did you space carbohydrates evenly throughout the day?	3.82 ± 2.04
How many days did you eat fish or other foods high in omega-3 fats?	0.76 ± 1.25
How many days did you eat foods that contained or were prepared with pure mustard oil, canola, walnut, olive, or flax oils?	3.93 ± 2.64
How many days did you not eat foods high in fat (such as high-fat dairy products, fatty meat, fried foods, or deep-fried foods)?	5.37 ± 1.95

## DISCUSSION

This study was carried out during the lockdown period in two tertiary care centres of western Nepal. The main finding of our study was poor glycemic control in Type 2 Diabetes Mellitus patients during COVID-19 lockdown among who there was low adherence to a specific diet and low physical activity level. Rodriguez-Perez C, et al. in their study, found higher adherence to the Mediterranean Diet, considered a healthy diet in the Spanish population during COVID-19 confinement.^9^ In contrast, we found a decrease in adherence to a healthy diet in the study population. We found increased consumption of sugary foods during the lockdown as in the studies reported by Ruiz-Roso M, et al.^10^ and Ghosh A, et al.^11^ Ingestion of animal protein enhances hepatic gluconeogenesis, stimulates glucagon secretion, thereby causing insulin resistance. On the other hand, plant-based foods increase insulin sensitivity.^12^ Decreased consumption of fruits, vegetables, foods with a low glycemic index, foods high in fibre consumption in our study population may account for poor glycemic control.

Mixed-effects of Covid-19 lockdown on physical activity have been reported from several studies around the world. Physical activity was decreased in the study reported by Sooriyaarachchi P, et al. from Srilanka,^13^ Zielinska M, et al. (Poland),^14^ Alshareef R, et al. in Saudi Arabia,^15^ and Giustin V, et al. in Sicilian population.^16^ However, physical activity was slightly increased in a study by Renzo D, et al. in Italy in body weight trainers,^17^ and no significant change in overall physical activity from a study by Kolokotroni O, et al. in Cyprus respectively.^18^

Physical activity has benefits in increasing insulin sensitivity and glycemic control in all ranges of the population, from children to older adults, including healthy people, prediabetes, and T2DM patients. During physical activity and for some hours after physical activity, increased skeletal muscle contraction leads to increased glucose uptake via facilitated diffusion through glucose transporter type four (GLUT4) in sarcolemma and T-tubules. This is because of the translocation of GLUT4 vesicles from intracellular sites to the plasma membrane through activation of intracellular signalling pathways brought by exercise.^19^ Possible reasons behind poor physical activity level in our study were strict measures imposed during lockdown with restriction of movement including morning walk, the closing of gym centres, yoga centres, and lack of usual office activities.

Disruption of glycemic control also has been reported in other studies. Khader M, et al. reported glycemic disruption in the Indian population, and the reasons for worsening glycemic control were decreased physical activity, increased food intake, and reduced frequency of clinical visits.^20^ However, the type of food intake is more important than the amount of food taken, which was not elucidated in this study. Likewise, during a lockdown, postprandial blood glucose level was significantly increased among T2DM patients in the study reported by Khare J, et al. in Central India^21^ and increased fasting blood glucose among T2DM patients in a study by Biamonte E, et al. in Italy.^22^ In contrast, a case-control study by D'onofrio L, et al. in Italy showed no significant impact of lockdown on glycemic control.^23^

Many factors might play a role in worsening glycemic control because glycemic control is determined by physical activity level, dietary adherence, frequency of self-monitoring of blood glucose,^24^ and medication adherence.^25^ Our findings support that the stricter measures applied to contain the coronavirus may adversely affect diabetes care, as depicted by Barone M, et al. in South and Central American countries.^26^

Most of the previous studies have reported an increase in fasting blood glucose or postprandial blood glucose. Still, it does not explicitly indicate the number of diabetes patients with controlled or uncontrolled blood glucose. Our study demonstrates the glycemic control (controlled as fasting plasma glucose <130 mg/dl and postprandial plasma glucose <180 mg/dl).^8^ We have quantified physical activity in MET min/week, including all aspects of daily living; carrying or lifting heavy loads, construction work, walking, running, cycling, playing games according to the standard questionnaire.^7^ We also have included the increased or decreased consumption of a specific type of food during lockdown as some foods are beneficial and others are harmful to the T2DM patients.^5^

There are few limitations in our study. We interviewed patients via telephone. It would have been better if we had been able to do face-to-face interviews showing them the flashcards with examples of physical activities to be used in the Global Physical Activity Questionnaire (GPAQ). However, as there was fear of contracting COVID-19 infection during the pandemic situation, a telephone interview was the best possible way to make them understand the questionnaire. We accept possible recall bias in this study. We have taken strict targets for glycemic control. However, individual patients may have varying glycemic goals due to their diabetes duration, age, comorbid conditions, cardiovascular disease, microvascular complications, and hypoglycemia unawareness.^8^

## CONCLUSIONS

Our study found that a majority of the study population with T2DM patients had poor glycemic control similar to the studies reported from other countries. There is poor adherence to diet and physical activity during the lockdown period. Also, the findings indicate the need for health care professionals to know the glycemic status in diabetic patients with a major focus on the importance of lifestyle changes and telemedicine services, especially during times like lockdown. Such study should be done in other parts of the country with a larger sample to determine the dietary adherence level and glycemic status in the usual time and lockdown. This might help to reduce the overall morbidity and mortality associated with complications due to poor glycemic control.
